# Early detection of urothelial premalignant lesions using hexaminolevulinate fluorescence cystoscopy in high risk patients

**DOI:** 10.1186/1479-5876-8-122

**Published:** 2010-11-22

**Authors:** Salvatore Blanco, Marco Raber, Biagio Eugenio Leone, Luca Nespoli, Marco Grasso

**Affiliations:** 1 Department of Urology, San Gerardo Hospital, University of Milano-Bicocca, Monza, Italy; 2 Department of Clinical Pathology, Desio Hospital, University of Milano-Bicocca, Monza, Italy; 3 Department of Surgery, San Gerardo Hospital, University of Milano-Bicocca, Monza, Italy

## Abstract

**Background:**

To evaluate fluorescence cystoscopy with hexaminolevulinate (HAL) in the early detection of dysplasia (DYS) and carcinoma in situ (CIS) in select high risk patients.

**Methods:**

We selected 30 consecutive bladder cancer patients at high risk for progression. After endoscopic resection, all patients received (a) induction BCG schedule when needed, and (b) white light and fluorescence cystoscopy after 3 months. HAL at doses of 85 mg (GE Healthcare, Buckinghamshire, United Kingdom) dissolved in 50 ml of solvent to obtain an 8 mmol/L solution was instilled intravesically with a 12 Fr catheter into an empty bladder and left for 90 minutes. The solution was freshly prepared immediately before instillation. Cystoscopy was performed within 120 minutes of bladder emptying. Standard and fluorescence cystoscopy was performed using a double light system (Combilight PDD light source 5133, Wolf, Germany) which allowed an inspection under both white and blue light.

**Results:**

The overall incidence was 43.3% dysplasia, 23.3% CIS, and 13.3% superficial transitional cell cancer. In 21 patients, HAL cystoscopy was positive with one or more fluorescent flat lesions. Of the positive cases, there were 4 CIS, 10 DYS, 2 association of CIS and DYS, 4 well-differentiated non-infiltrating bladder cancers, and 1 chronic cystitis. In 9 patients with negative HAL results, random biopsies showed 1 CIS and 1 DYS. HAL cystoscopy showed 90.1% sensitivity and 87.5% specificity with 95.2% positive predictive value and 77.8% negative predictive value.

**Conclusion:**

Photodynamic diagnosis should be considered a very important tool in the diagnosis of potentially evolving flat lesions on the bladder mucosa such as DYS and CIS. Moreover, detection of dysplasic lesions that are considered precursors of CIS may play an important role in preventing disease progression. In our opinion, HAL cystoscopy should be recommended in the early follow-up of high risk patients.

## Introduction

Bladder cancer is costly in both human and societal terms, yet the level of awareness of the disease and its early symptoms is low among the public and health care professionals. There is also a poor understanding of the potential causative role played by exposure to workplace carcinogens [1].

Transitional cells cancer is the most common bladder neoplasm and his infiltrating form may heavily affect the patient survival. In this regard the main challenge is to early diagnose aggressive cancer yet in a limited stage or better while it has not became infiltrating. A part of superficial bladder cancers indeed may recur even several times after primary resection without showing any worsening in their malignant potential. In some cases they come through the lamina propria, the deep submucosa and muscular wall showing a clear infiltrating course. Unfortunately the biological reasons of this radical changing of tumor behaviour are not well understood. However, the presence of non-papillary carcinoma in situ (CIS) is really considered a source of invasive bladder cancer [2]. Furthermore, it has been documented that even in patients with papillary disease, most invasive cancers develop from adjacent areas of carcinoma in situ [3]. However, in order to modify the natural history of bladder cancer an earlier diagnosis might be done by identification of a known precursor of CIS called severe dysplasia (DYS) [4,5]. Dysplasia is often located in normal-appearing bladder mucosa and can be easily missed under standard white-light (WL) cystoscopy [6]. Voided-urine cytology has proven useful as a non-invasive adjunct in the detection of CIS, although its sensitivity in the detection of DYS may be questionable [7,8].

Several investigators have used photodynamic agents to detect dysplastic urothelium [9,10]. Zaak et al previously concluded that 5-aminolevulinic acid (5-ALA) provided the most efficient diagnostic agent for patients with flat, high-risk urothelial lesions (CIS and DYS) compared with WL cystoscopy and cytology [11]. In our study, we used a recently introduced, more lipophylic ester of 5-ALA, hexaminolevulinate (HAL) to study DYS and CIS, and compared the detection rate with this agent to that of classic WL cystoscopy and fluorescence cystoscopy in select high risk patients.

## Materials and methods

Between March 2007 and February 2008, 30 consecutive bladder cancer patients at high risk for progression were selected. Patients if needed started a BCG induction schedule within 30 days (once weekly for 6 weeks). The following WL and HAL cystoscopy control was performed after 3 months in order to minimize the likelihood of false positives [12,13]. Patients with porphyria, gross haematuria, acute urinary tract infection, multi-drug allergies, and women not on adequate contraceptive measures or who were breast feeding were excluded [14].

HAL at doses of 85 mg (GE Healthcare, Buckinghamshire, United Kingdom) dissolved in 50 ml of solvent to obtain an 8 mmol/L solution was instilled intravesically with a 12 Fr catheter into an empty bladder and left for 90 minutes. The solution was freshly prepared immediately before instillation. Cystoscopy was performed within 120 minutes of bladder emptying as described below.

The surface of the bladder absorbs the HAL solution and converts it to the endogenous pigment, protoporphyrin IX. This pigment is selectively deposited in the tumour and causes fluorescence in the red-range when excited by blue-violet light. The comparison of HAL with standard cystoscopy was performed using a within-patient design by inspecting the bladder under WL first, followed by blue light (fluorescence). Because cystoscopy was combined with the immediate resection of suspicious lesions, all patients received sedation or spinal anaesthesia. Before endoscopic inspection, the bladder was evacuated. Standard and fluorescence cystoscopy was performed using a double light system (Combilight PDD light source 5133, Wolf, Germany) which allowed an inspection under both white and blue light.

The purpose of preliminary WL cystoscopy was to identify and note any exophytic lesions and suspicious areas in the bladder chart. Subsequently, under blue light cystoscopy, we aimed to determine the number and location of all fluorescing areas on the same bladder chart. In patients without suspicion, 5 random biopsies were taken from normal appearing urothelium.

All biopsies and resected materials were analyzed by a single pathologist blinded to the fluorescence cystoscopy results. Lesions were staged and graded according to the 2004 WHO classification [15].

Safety assessments, including physical examinations, vital signs, and blood sampling for hematology and biochemistry were performed at baseline and again 24 hours after HAL instillation. All spontaneously reported and observed adverse events were documented during the hospital stay. Patients were followed for roughly 10 days until the consultation of their histologic results and were interviewed for any adverse effects after hospital discharge.

Categorical data were examined by chi-square test, while continuous variables were evaluated by the t-test. Specificity, sensitivity, positive predictive value (PPV), and negative predictive value (NPV) were calculated with the usual mathematical formulas.

## Results

Of the 30 patients, 24 were males and 6 were females. Their mean age was 67 (SD, 7.8; range, 46-76) years. For 11 patients, high risk transitional cancer was the first episode while in the remaining patients high risk episode was recurrent. (range, 2-11 resections; average 2.8 resections). In all patients, WL cystoscopy was negative. Urinary cytology was positive in 9 patients and suspected in 4 cases.

The overall incidence of DYS was 43.3% (13/30), CIS was 23.3% (7/30), and superficial transitional cell cancer was 13.3% (4/30). Disease-free follow-up occurred in 26.7% (8/30) of patients. In 21 patients, the HAL cystoscopy was positive, with one or more fluorescing flat lesions present (mean ± SD, 2.7 ± 1.4; range 1-5). The positive cases consisted of 4 CIS, 10 DYS, and 2 associations of CIS and DYS, well-differentiated superficial bladder cancer non-infiltrating to the lamina *propria *in 4 cases, and chronic cystitis in 1 case. In 9 patients with negative results by HAL, the 6 random biopsies showed one case each of CIS and DYS. HAL cystoscopy showed 90.1% sensitivity (95% CI, 0.53-0.87) and 87.5% specificity (95% CI, 0.47-0.99) and 95.2% PPV (95% CI, 0.74-0.99) and 77.8 NPV (95% CI, 0.40-0.96). CIS and DYS were both visible as a brilliant-red, well-limited fluorescence area in contrast with the normal adjacent urothelium.

HAL fluorescence cystoscopy was well tolerated and no unexpected events were reported.

## Discussion

Bladder cancer risk categories are based on clinical and histopathologic parameters such as number of tumours, tumour size, prior recurrence rate, T category, presence of concomitant CIS, and tumour grade [16,17]. Among these, CIS is considered an important risk factor for disease progression because specific survival is heavily affected by the presence of CIS alone or associated with papillary superficial bladder cancer and non papillary T1 tumours [18]. So it should be necessary an earlier diagnosis when mucosal changes are still precursor of CIS. DYS is considered an epithelial abnormality appearing as a flat lesion on the bladder mucosa and a precursor of CIS [19]. This premalignant lesion might have important implications in the early diagnosis of bladder cancer progression. Several recent studies have shown that concomitant or single DYS is associated with a considerable risk for disease progression [20-22]. However, diagnosis is very difficult because, in the early stages, both lesions are indistinguishable from the normal-appearing bladder mucosa [6] and urine cytology testing might not be sufficiently sensitive [23].

The situation can be significantly improved with the use of photo sensitizers, e.g. 5-ALA or HAL, which can be safely administered intravesically and make these flat lesions visible within an otherwise normal bladder mucosa. Our results confirm the advantage in the diagnosis of potentially evolving flat lesions (DYS and CIS) on the bladder mucosa examined by photodynamic, rather than classic WL, cystoscopy. A real benefit was shown in the diagnosis of early papillary superficial bladder tumours that were not yet visible, confirming previous observations [24-28].

Regarding dysplasia, in a previous study, Zaak et al. concluded that photodynamic diagnosis using 5-ALA was an efficient diagnostic technique for patients with flat, high-risk urothelial lesions compared with classic WL cystoscopy and cytology [11]. In our study, we used HAL, a potent ester of aminolevulinic acid, that provides better selectivity, brighter fluorescence, and requires a shorter instillation time [29,30].

Another point of discussion is the incidence of DYS and CIS, which was 43.3% and 23.3%, respectively, in our study. This means that 66.6% of our patients had a potential evolving flat lesion. In the absence of photodynamic diagnosis, the incidence of such lesions would have been only 6.7%. These results suggest the careful consideration of all therapeutic possibilities, beginning with the careful endoscopic resection, as well as the therapeutic effect of immunoprophylaxis in these high risk patients. Because bladder cancer is often multicentric, particularly when it is of high grade, a standard WL resection might miss invisible tumours. Moreover, a BCG induction schedule might be not sufficient to treat these lesions making treated indeed during maintenance schedule. However, the detection rate of these otherwise undiagnosed lesions is higher with photodynamic screening.

The limitations of this study are the small number of patients included. However, we feel that this limitation is balanced by the highly selected series.

Further studies are needed to determine whether this important and not inexpensive diagnostic tool must be reserved for primary or secondary look resections of high risk patients and if the improvement in the rate of detection of flat lesions in the follow-up may improve the use of additional treatment and the prognosis of these patients.

## Conclusions

Photodynamic diagnosis should be considered a very important tool in the diagnosis of potentially evolving flat lesions on the bladder mucosa such as DYS and CIS. Moreover, detection of dysplasic lesions that are considered precursors of CIS may play an important role in preventing disease progression. In our opinion, HAL cystoscopy should be recommended in the early follow-up of high risk patients.

## Competing interests

The authors declare that they have no competing interests.

## Authors' contributions

SB has conceived the study and participated in its draft and design. MR has participated in its design and draft. BEL has carried out the histological analysis. LN has participated in its revision. MG has conceived the study and participated in its design and coordination. All authors read and approved the final manuscript.

## References

[B1] Grasso M Bladder Cancer: a major public health issue Eur Urol Suppl 2008 7 510 5 10.1016/j.eursup.2008.04.001

[B2] Herr HW Natural history of superficial bladder tumors: 10- to 20-year follow-up of treated patients World J Urol 1997 15 84 10.1007/BF02201977 9144896

[B3] Koss LG Mapping of the urinary bladder: its impact on concepts of bladder cancer Hum Patol 1979 10 533 548 10.1016/S0046-8177(79)80097-0 527959

[B4] Murphy WM Busch C Algaba F Intraurothelial lesions of urinary bladder: morphologic considerations Scand J Urol Nephrol 2000 205 Suppl 67 81 11144906

[B5] Mostofi FK Davis CJ Sesterhenn I Histological typing of urinary bladder tumours World Health Organization international histological classification of tumours 1999 Berlin: Springer

[B6] Soloway MS Murphy W Rao MK Cox C Serial multiple-site biopsies in patients with bladder cancer J Urol 1978 120 57 59 671606 10.1016/s0022-5347(17)57037-8

[B7] Murphy WM Current status of urinary cytology in the evaluation of bladder neoplasms Hum Pathol 1990 21 886 896 10.1016/0046-8177(90)90171-Z 2203672

[B8] Bastacky S Ibrahim S Wlczynski SP Murphy WM The accuracy of urinary cytology in daily practice Cancer 1999 87 118 128 10.1002/(SICI)1097-0142(19990625)87:3<118::AID-CNCR4>3.0.CO;2-N 10385442

[B9] Kriegmair M Knuchel R Stepp H Hofstaedter F Hofstetter A Detection of early bladder cancer by 5-aminolevulinic acid induced porphyrin fluorescence J Urol 1996 155 105 110 10.1016/S0022-5347(01)66559-5 7490803

[B10] Jichlinski P Forrer M Mizeret J Clinical evaluation of a method for detecting superficial surgical transitional cell carcinoma of the bladder by light-induced fluorescence of protoporphyrin IX following the topical application of 5-aminolevulinic acid: preliminary results Lasers Surg Med 1997 20 402 408 10.1002/(SICI)1096-9101(1997)20:4<402::AID-LSM5>3.0.CO;2-U 9142679

[B11] Zaak D Hungerhuber E Schneede P Stepp H Frimberger D Corvin S Schmeller N Kriegmair M Hofstetter A Knuechel R Role of 5-Aminolevulinic Acid in the Detection of Urothelial Premalignant Lesions Cancer 2002 95 2580 10.1002/cncr.10821 12216090

[B12] Colombo R Naspro R Bellinzoni P Photodynamic diagnosis for follow-up of carcinoma in situ of the bladder Ther Clin Risk Manag 2007 3 6 1003 7 18516260 PMC2387289

[B13] Grimbergen MC van Swol CF Jonges TG Reduced specifi city of 5-ALA induced fluorescence in photodynamic diagnosis of transitional cell carcinoma after previous intravesical therapy Eur Urol 2003 44 1 51 6 10.1016/S0302-2838(03)00210-0 12814675

[B14] Schmidbauer J Witjes F Schmeller N Donat R Susani M Marberger M Hexvix PCB301/01 Study Group Improved detection of urothelial carcinoma in situ with hexaminolevulinate fluorescence cystoscopy J Urol 2004 171 1 135 8 10.1097/01.ju.0000100480.70769.0e 14665861

[B15] Algaba F Amin M Eble JN, Sauter G, Epstein JI, Sesterhenn I Tumours of the urinary system: non invasive urothelial neoplasias WHO classification of tumours of the urinary system and male genital organs 2004 Lyon: IARCC Press

[B16] Millan-Rodriguez F Chechile-Toniolo G Salvador-Bayarri J Palou J Algaba F Vicente-Rodriguez J Primary superficial bladder cancer risk groups according to progression, mortality and recurrence J Urol 2000 164 3 Pt 1 680 684 10.1016/S0022-5347(05)67280-1 10954628

[B17] Sylvester RJ van der Meijden AP Oosterlinck W Witjes A Bouffioux C Denis L Newling DW Kurth K Predicting recurrence and progression in individual patients with stage TaT1 bladder cancer using EORTC risk tables: a combined analysis of 2596 patients from seven EORTC trials Eur Urol 2006 49 3 466 477 10.1016/j.eururo.2005.12.031 16442208

[B18] Fukui I Yokokawa M Sekine H Carcinoma in situ of the urinary bladder. Effect of associated neoplastic lesions on clinical course and treatment Cancer 1987 59 164 73 10.1002/1097-0142(19870101)59:1<164::AID-CNCR2820590132>3.0.CO;2-Z 3791145

[B19] Lopez-Beltran A Cheng L Anderson L Preneoplastic non-papillary lesions and conditions of the urinary bladder: an update based on the Ancona international consultation Virchows Arch 2002 440 3 11 10.1007/s00428-001-0577-6 11942574

[B20] Althausen AF Prout GR Daly JJ Non-invasive papillary carcinoma of the bladder associated with carcinoma in situ J Urol 1976 116 575 580 978809 10.1016/s0022-5347(17)58916-8

[B21] Kiemeney LA Witjes JA Heijbroek RP Debruyne FM Verbeek AL Dysplasia in normal-looking urothelium increases the risk of tumour progression in primary superficial bladder cancer Eur J Cancer 1994 30A 1621 1615 10.1016/0959-8049(94)E0133-O 7833133

[B22] Cheng L Cheville JC Neumann RM Bostwick DG Flat intraepithelial lesions of the urinary bladder Cancer 2000 88 625 631 10.1002/(SICI)1097-0142(20000201)88:3<625::AID-CNCR20>3.0.CO;2-A 10649257

[B23] Cheng L Chevilee JC Neumann RM Bostwick DG Natural history of urothelial dysplasia of the bladder Am J Surg Pathol 1999 23 443 447 10.1097/00000478-199904000-00009 10199474

[B24] Grossman HB Improving the management of bladder cancer with fluorescence cystoscopy J Environ Pathos Toxicol Oncol 2007 26 143 7 10.1615/jenvironpatholtoxicoloncol.v26.i2.90 17725540

[B25] Fradet Y Grossman HB Gomella L A comparison of hexaminolevulinate fluorescence cystoscopy and white light cystoscopy for the detection of carcinoma in situ in patients with bladder cancer: a phase III, multicenter study J Urol 2007 178 68 73 10.1016/j.juro.2007.03.028 17499291

[B26] Grossman HB Gomella L Fradet Y A phase III, multicenter comparison of hexaminolevulinate fluorescence cystoscopy and white light cystoscopy for the detection of superficial papillary lesions in patients with bladder cancer J Urol 2007 178 62 7 10.1016/j.juro.2007.03.034 17499283

[B27] Jichlinski P Guillou L Karlsen SJ Hexyl aminolevulinate fluorescence cystoscopy: new diagnostic tool for photodiagnosis of superficial bladder cancer - a multicenter study J Urol 2003 170 226 9 10.1097/01.ju.0000060782.52358.04 12796694

[B28] Jocham D Witjes F Wagner S Zeylemaker B van Moorselaar J Grimm MO Muschter R Popken G König F Knüchel R Kurth KH Improved detection and treatment of bladder cancer using hexaminolevulinate imaging: a prospective, phase III multicenter J Urol 2005 174 3 862 6 10.1097/01.ju.0000169257.19841.2a 16093971

[B29] Kloek J Akkermans W Beijersbergen van Henegouwen GM Derivatives of 5 aminolevulinic acid for photodynamic therapy: enzymatic conversion into protoporphyrin Photochem Photobiol 1998 67 150 10.1111/j.1751-1097.1998.tb05178.x 9477773

[B30] Marti A Lange N van den Bergh H Sedmera D Jichlinski P Kucera P Optimisation of the formation and distribution of protoporphyrin IX in the urothelium: an in vitro approach J Urol 1999 162 546 10.1016/S0022-5347(05)68625-9 10411086

